# Aberrant DNA methylation of alternative promoter of DLC1 isoform 1 in meningiomas

**DOI:** 10.1007/s11060-016-2261-3

**Published:** 2016-09-10

**Authors:** M. Bujko, P. Kober, N. Rusetska, M. Wakuła, K. Goryca, E. Grecka, E. Matyja, J. Neska, T. Mandat, W. Bonicki, J. A. Siedlecki

**Affiliations:** 1 Department of Molecular and Translational Oncology, Maria Sklodowska-Curie Memorial Cancer Center and Institute of Oncology, 5, W.K. Roentgena, 02-781 Warsaw, Poland; 2 Department of Genetics, Maria Sklodowska-Curie Memorial Cancer Center and Institute of Oncology, Warsaw, Poland; 3 Department of Experimental and Clinical Neuropathology, M. Mossakowski Medical Research Centre, Polish Academy of Sciences, Warsaw, Poland; 4 Department of Immunology, Maria Sklodowska-Curie Memorial Cancer Center and Institute of Oncology, Warsaw, Poland; 5 Department of Neurosurgery, Maria Sklodowska-Curie Memorial Cancer Center and Institute of Oncology, Warsaw, Poland

**Keywords:** DNA methylation, Alternative promoter, *DLC1*, RHO-GTPases, Meningioma, Epigenetic, Expression

## Abstract

DLC1 encodes GTPase-activating protein with a well-documented tumor suppressor activity. This gene is downregulated in various tumors through aberrant promoter hypermethylation. Five different DLC1 isoforms can be transcribed from alternative promoters. Tumor-related DNA methylation of the *DLC1* isoform 1 alternative promoter was identified as being hypermethylated in meningiomas in genome-wide DNA methylation profiling. We determined the methylation pattern of this region in 50 meningioma FFPE samples and sections of 6 normal meninges, with targeted bisulfite sequencing. All histopathological subtypes of meningiomas showed similar and significant increase of DNA methylation levels. High DNA methylation was associated with lack of DLC1 protein expression in meningiomas as determined by immunohistochemistry. mRNA expression levels of 5 isoforms of DLC1 transcript were measured in an additional series of meningiomas and normal meninges. The *DLC1* isoform 1 was found as the most expressed in normal control tissue and was significantly downregulated in meningiomas. Transfection of KT21 meningioma cell line with shRNA targeting DLC1 isoform 1 resulted in increased activation of RHO-GTPases assessed with pull-down assay, enhanced cell migration observed in scratch assay as well as slight increase of cell metabolism determind by MTT test. Results indicate that isoform 1 represents the main pool of DLC1 protein in meninges and its downregulation in meningiomas is associated with hypermethylation of CpG dinucleotides within the corresponding promoter region. This isoform is functional GAP protein and tumor suppressor and targeting of its expression results in the increase of DLC1 related cell processes: RHO activation and cell migration.

## Introduction

Meningiomas are among the most frequent primary intracranial tumors in adults. Most are benign (WHO grade I) neoplasms and only some transform to more aggressive subtypes (grade II or III) [1]. They represent an important clinical problem, as tumor recurrence is observed in approximately 20 % of all patients and in 30–40 % of those with incompletely resected tumors [2]. Standard treatment involves surgical resection with optional radiosurgery or radiotherapy in cases of incomplete resection. Despite the fact that meningiomas are common intracranial tumors, relatively little is known about their molecular pathogenesis as compared to other tumors diagnosed with similar frequency. Some important tumor driver genes have been identified including the most common *NF2* or *TRAF7*, but the role of epigenetic aberrations is still poorly understood.

The *DLC1* (*Deleted in Liver Cancer 1*) gene encodes GTPase-activating protein (GAP) belonging to the RhoGAP protein family. These proteins act as molecular switches through the regulation of small GTP-binding proteins. RhoGAPs take part in cell signaling and are involved in regulating a variety of cellular proteins [3].

DLC1 is well described as a tumor suppressor that prevents cell migration, invasion and metastatic progression in various cancer types [4, 5]. The expression of this gene was shown to be notably reduced in various tumors. Genomic mutations and aberrant promoter methylation have also been proved to be implicated in gene silencing. In some cancers, the loss of *DLC1* expression was shown to be of prognostic value [4, 6].

**Fig. 1:**
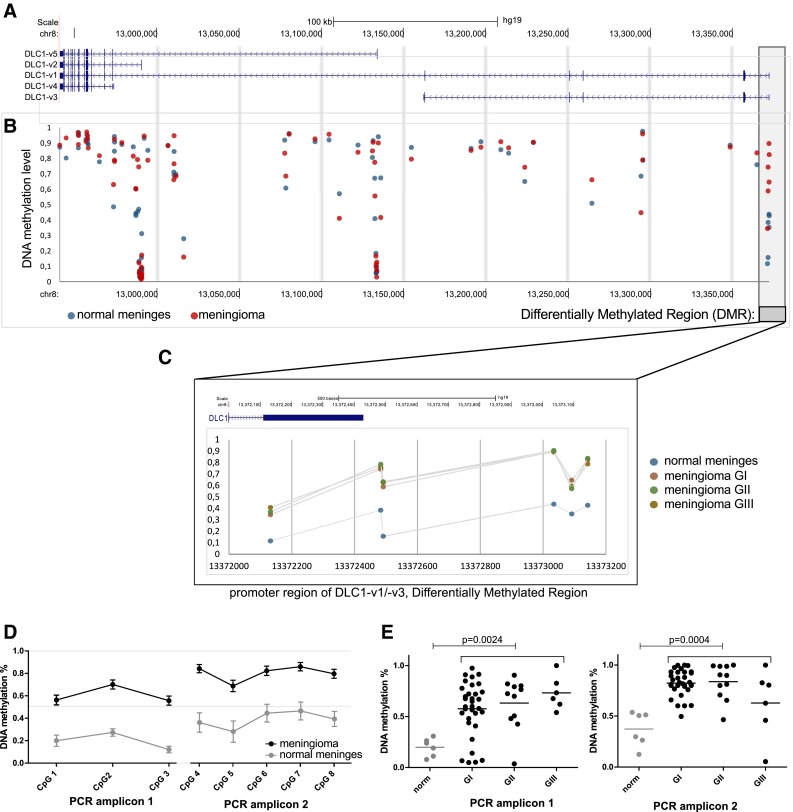
**a** Genomic location of 5 transcription variants of DLC1. **b, c** DNA methylation level of the *DLC1* locus at CpGs measured by the HumanMethylation 450K array (Illumina) in meningiomas and normal meninges. Each *blue*/*red dot* represents an individual CpG. **d** DNA methylation levels in meningiomas of different grade at CpGs within differentially methylated regions (DMR) (HumanMethylation 450K microarray data). **d, e** DNA methylation level of DMR at 5′ region of DLC1 locus determined by targeted bisulfite sequencing of two PCR amplicons. **d** Comparison of average methylation levels of each CpGs in meningiomas and normal samples. Each *dot* represents the mean value and *error bars* represent standard errors of the mean. **e** Comparison of average DNA methylation levels for two amplicons in normal meninges and different grade meningiomas. *Horizontal lines* denote the mean value

An important issue not considered previously in most cancer studies on DLC1, is that multiple transcript variants of this gene have been identified. Five different transcripts are encoded in the *DLC1* locus which are expressed through alternative promoters and undergo alternative splicing (Fig. 1a).

Interestingly, the different short *DLC1* alternative variants are generated from different parts of the gene body and encode varying protein contents.

Alternative DLC1 variants have been shown to be expressed in humans and mice [7–9] However, various human tissues show different levels of mRNA in individual variants [7]. Alternative promoters of different transcript isoforms seem to be regulated epigenetically [7]. The vast majority of previous research on *DLC1’s* role in cancer have not addressed the issue of multiple transcript variants or were only focused on *DCL1*’s role in variant 2. Expression of different alternative *DLC1* transcripts was considered only in a very few cancer studies [7, 9].

*DLC1* downregulation in meningiomas was reported once in a study on a small series of samples; 6 tumor samples and 4 normal meninges, however, no DNA methylation of the gene promoter have been found [10]. It is also known that *DLC1* mutations have not been identified in meningiomas for whole exome sequencing studies and no mutations of this gene have been reported in the COSMIC database [11, 12].

In contrast to the lack of reported *DLC1* promoter methylation in meningiomas, we identified 5′ region of *DLC1* as being frequently hypermethylated in this tumor. This prompted us a deeper investigation of DNA *DLC1* promoter methylation and its possible role in gene expression.

The presented study numbers *DLC1* transcription variants according to the Refseq database using sequence accession numbers as follows; *DLC1* variant 1 (*DLC1-v1*)-NM_182643, variant 2 (*DLC1-v2*)-NM_006094, variant 3 (*DLC1-v3*)-NM_024767, variant 4 (*DLC1-v4*)-NM_001164271.1 and variant 5 (*DLC1-v5*)-NM_001316668 [13].

## Materials and methods

### Patients and tissue samples

Archival tissue meningiomas samples from patients who underwent surgical tumor resection at the Cancer Center and Institute of Oncology in Warsaw were used for DNA isolation and genomic sequencing. Fifty patients with sporadic meningiomas (33 WHO grade I, 11 grade II and 6 grade GIII) were enrolled in the study. All tissue samples were histopathologically examined involving a review of diagnosis and selecting representative tissue section suitable for molecular analysis. Patients’ characteristics are listed in Table 1. Control samples of meninges were harvested at non-neurooncological neurosurgical procedures while duroplasty was an element of those procedures.

**Table 1:** Patients characteristics

Patients	55
Male	18
Female	32
Age (years)	
Range	26–86
Median	56
WHO meningioma subtype	
WHO grade I	33
Transitional	12
Meningothelial	11
Fibrous	5
Psammomatous	3
Microcystic	1
Metaplastic	1
WHO grade II atypical	11
WHO GIII anaplastic	6
Tumor location	
Skull base	20
Frontal convexity	5
Cereblar falx	4
Parietal convexity	4
Sphenoid wing	3
Tuberculum sellae	3
Fronto-parietal convexity	2
Parasaggital	2
Petroclival	2
Anterior fossa	1
Cerebellar tentorium	1
Orbit	1
Spinal canal	1
Temporal convexity	1

Formalin-fixed and paraffin-embedded (FFPE) tissue samples were manually macrodissected where DNA was isolated using a QIAmp DNA mini kit (Qiagen). In order to isolate high quality RNA for qRT-PCR analysis, 16 meningioma Sections (15 GI, 1 GII tumors) and 4 normal meninges sections were collected and snap frozen in liquid nitrogen. Total RNA was isolated using the RNeasy Mini (Qiagen).

Approval was obtained from the Institutional Ethics Committee for experimenting on human patient samples.

### Genome-wide DNA methylation analysis

The HumanMethylation 450K Infinium Methylation BeadChip (Illumina) was used to profile whole-genome DNA methylation of 8 benign (GI), 5 atypical (GII) and 4 anaplastic meningiomas (GIII) as well as 4 normal meningeal samples. Laboratory procedures were performed by the AROS Applied Biotechnology service. Genomic DNAs were bisulfite-converted, using the EZ-96 DNA Methylation kit (Zymo Research, Orange, CA, USA). Probes with missing intensity signals were discarded (9541 probes out of 485, 578). Probes were divided into design classes and annotated to genome location according to the lluminaHumanMethylation450k. db library. Intensities were normalized with the BMIQ package (version 1.3) using default parameters [14]. Probe mapping to differentially methylated sites were selected according to the P value from the *t* test (Welch variant) after correction for multiple hypothesis testing with the Benjamini–Hochberg algorithm.

### DNA methylation analysis with targeted bisulfite sequencing

Primers for targeted bisulfite sequencing of two *DLC1* promoter regions were designed and validated by Zymo Research and were synthesized by Integrated DNA Technologies (IDT). The primers’ quality control (QC) tested for specific amplification of 1 ng bisulfite-treated DNA (in duplicate) using converted DNA QC criteria, which included robust and specific amplification of the bisulfite primers on real-time PCR (Roche LC 480; C_t_ values less than 40 cycles and CV < 10 % for duplicates). DNA samples were bisulfite treated with the EZ DNA Methylation-Direct™ Kit. Targeted sequencing was done using the Fluidigm Access Array with Zymo Research’s Targeted sequencing service protocol. Sample loading, harvesting, and pooling was performed according to the manufacturer’s protocol (Fluidigm). Amplicons were pooled, diluted 1:100 and then amplified using barcoded adaptor-linkers from Fluidigm, according to manufacturer’s protocols. Reactions were cleaned up using the DNA Clean and Concentrator™-5 and products were normalized by concentration and then pooled. The library was denatured, diluted, and sequenced on the Illumina MiSeq according to the manufacturer’s protocols. The sequencing run was a 150-base paired-end run. Two amplicons for *DLC1* promoter were analyzed: amplicon 1 (chr8:13372333–13372641, hg19) and amplicon 2 (chr8:13372933–13373241, hg19).

### Quantitative PCR expression assessment (qRT-PCR)

1 µg of each RNA sample was subjected to reverse transcription with the Transcriptor First Strand cDNA Synthesis Kit (Roche). qPCR was undertaken using the 384 well format and Applied Biosystems 7900HT Fast Real-Time PCR System (Applied Biosystems) with the Sensimix SYBR kit (Bioline), according to manufacturer’s recommendations in a volume of 5 µl. *RPL32* was used as reference, as this gene was previously identified as the most stable for meningiomas and normal arachnoid [15]. Standard curves based on amplification of known cDNA template concentrations were used for calculating PCR efficiency. Because we were not able to design an effective qRT-PCR primer specific for DLC-v1 exclusively, we used qRT-PCR primers specific for both *DLC1-v1* and *DLC1-v3*. The expression of *DLC1-v1* was calculated by the subtraction of the result obtained with primers unique for *DLC1-v3* from the result obtained with the primers common for variant 1 and 3. The PCR primers’ sequences are presented in Table 2.

**Table 2:** Sequences of qRT-PCR primers and oligonucleotides encoding shRNA cassette targeting DLC1-v1/-v3 sequence

PCR primers			
DLC1 variant	Forward 5′→3′	Reverse 5′→3′	Amplicon length (bp)
*DLC1-v1/DLC1-v3*	GCGCCCTATCTCGATCTTCT	ACAATTAAAGGAGACCCTGGC	109
*DLC1-v2*	CTTCCCCACAGCGCTTC	ACAAGCTTCCTTGGCTTCAA	98
*DLC1-v3*	CTTCCACTCCAGTAGCCAAT	GCGAGAAAACAGAACCAAAATG	95
*DLC1-v4*	TGATCACGCAACAGTGAAACA	ATCGATGGGGAACAGGAAAT	120
*DLC1-v5*	AGCGATCACATCAGGGACTC	CCAATCACAAGCTTCCTTGG	105

Oligonucleotides with shRNA cassette			
Sense strand	GATCGGCACCTGAGAAACAATTGCTTAACTTCAAGAGAGTTAAGCAATTGTTTCTCAGGTGCTTTTTTGA*		
Complementary strand	AGCTTCAAAAAAGCACCTGAGAAACAATTGCTTAACTCTCTTGAAGTTAAGCAATTGTTTCTCAGGTGCC*		

*DLC1 complementary sequence is underlined

### Immunohistochemistry (IHC)

Immunohistochemical staining was performed on 4 μm formalin-fixed, paraffin-embedded tissue sections of 18 meningiomas (5 meningothelial GI, 4 fibrous GI, 4 atypical GII and 5 anaplastic GIII tumors) using the Envision Detection System and DAB reagent (DAKO). Sections were deparaffinized with xylene and rehydrated in a series of ethanol solutions decreasing in concentration. Heat-induced epitope retrieval was carried out in a Target Retrieval Solution pH 6 (DAKO) in a 96 °C water bath for 20 min. For cooling down, slides in retrieval solutions were kept at room temperature for 30 min and they were treated for 5 min with an Endogenous Peroxidase Blocker (DAKO). Slides were then incubated with polyclonal antibody against DLC1 (PA5-28443, Pierce/Thermo Scientific) at 1:250 dilution for 60 min at room temperature and subsequently labelled with the Envision Detection System (DAKO). This antibody was produced using immunogen peptide corresponding to a region within amino acids 1 and 43 of Human DLC1-v1. Therefore it recognizes two protein isoforms: DLC1-v1 (170 kDa) and DLC1-v3 (51 kDa) and do not detect the remaining DLC1 variants lacking N-terminal sequence used for immunization. The colour reaction product was developed with 3,3′-diaminobenzidine, tetrahydrochloride (DAB) (DAKO) as substrate. Tissues were then counterstained with Mayer’s haematoxylin, dehydrated and cover-slipped using a permanent mounting solution (Toluene-Free Mounting Medium, Dako). Positive and negative controls were included. Staining intensity was assessed by a four-grade scale: 0—lack of expression, 1—weak expression, 2—moderate expression and 3—strong expression. A section of normal colonic mucosa sample was used as the technical positive control. The stained tissue slides were examined by the investigator blinded to the DNA methylation results.

### Western blotting

Cells were lysed in ice cold RIPA buffer, incubated for 30 min in 4 °C and centrifuged at 12,500 rpm for 20 min at 4 °C. Samples were resolved using SDS-PAGE and electrotransferred to polyvinylidene fluoride membranes (PVDF) (Pierce/Thermo Fisher). DLC1 was detected with polyclonal anti-DLC1 antibody (PA5-28443, Pierce/Thermo Fisher), and secondary anti-rabbit antibody conjugated to HRP (#7074, Cell Signalling). α-tubulin detected with polyclonal rabbit antibody (#2125, Cell Signalling) or β-actin detected with mouse HRP-conjugated antibody (#12262 Cell Signalling) served as control. Visualization was performed with the enhanced chemiluminescence method using WesternBright Quantum (Advansta, Menlo Park, CA, USA) and CCD digital imaging system Alliance Mini HD4 (UVItec Limited, Cambridge, United Kingdom).

### Silencing of DLC1 in KT21 meningioma cell line

KT21 meningima cells were used for DLC1 silencing and in vitro assays. The cells were grown on DMEM medium supplemented with 10 % FBS (Life Technologies) at 37 °C and 5 % CO_2_ concentration.

Silencing of DLC1v1/v3 was achieved with the use of the pGFP-B-RS vector (OriGene Technologies, Inc., Rockville, MD, USA) encoding shRNAs complementary to 5′ regions of DLC1-v1 mRNA. Commercially available universal plasmid with scrambled shRNA cassette (sh control): pGFP-B-RS-scrambled Non-Effective (OriGene Technologies, Inc., Rockville, MD, USA) served as a negative control (see Table 1 for details). The shRNA hairpin complementary to 25 bp at 5′ fragment of DLC1-v1 was designed using siRNA Wizard v3.1 (http://www.invivogen.com/sirnawizard/) and designed oligonucleotides included BamHI and HindIII restriction sites for cloning. Synthetic 78 bp-long single-stranded oligonucleotides were annealed into double-stranded DNA insert and cloned into pGFP-B-RS vector, digested with BamHI and HindIII, according to manufacturers’ recommendations. The construct sequence was verified with Sanger sequencing. Sequences of oligonucleotides are provided in Table 2.

The cells at 70–80 % confluence were transfected using Lipofectamine 2000 (Life Technologies), according to the manufacturer’s instructions and harvested or assayed after culturing for 48 h. To assess transfection efficacy and to test the effect on DLC1-v1 expression transfected cells were analyzed and sorted using flow cytometer with cell sorter FACS ARIA III (BD biosciences).

### In vitro assays

MTT assay, determining cell metabolic activity was performed according to standard protocol. Briefly, cells were seeded onto 96-well plates in triplicates, transfected with shRNA encoding pGFP-B-RS vector or sh control vector and cultured for 48 h followed by MTT addition. After 4 h of incubation, 500 μL of DMSO was added to each well and absorbance intensity was recorded at 595 nm on Victor 3 microplate reader (Perkin Elmer, Wellesley, MA, USA).

Scratch wound healing assay was performed based on previously published protocol. Cells were seeded onto 10 cm plates, transfected with plasmid vectors at 90 % confluence and cultured for 48 h. Scratch wounds were done by scraping the cell layer across each culture plate using the tip of 10 μl pipette. 10 fields were chosen, marked on the plates, visualized and captured by microscopy (magnification ×40). The same 10 fields were captured after 16 h incubation. The cell migration distance was measured at each of 10 fields at the marked positions with the use of Quick Photo Camera 2.3 software (Promicra, Prague, Czech Republic). The migration distance was calculated as the difference between initial scratch width and width of cell free area after 16 h culturing at the marked positions.

### Active RHO-GTPases pull down assay

The status of RHO-GTPases activation was assessed using Active Rho Pull-Down and Detection Kit (Thermo/Pierce), according to manufacturer’s protocol.

Cells were seeded onto four 10 cm plates and transfected with one of the plasmid vectors (encoding shRNA targeting DLC1-v1 or shRNA control) in duplicates. 48 h after transfection the cells were washed with ice cold TBS, and lysed with cell lysis buffer. Each lysate was pooled from two 10 cm plates and centrifuged at 10,000 rpm for 2 min at 4 °C. Equal amounts of protein extracts were used for pull-down assay and incubated for 1 h at 4 °C with Rhotekin-RBD protein Sepharose beads. Pellets were washed three times with 0.4 ml of Lysis/Binding/Wash buffer and eluted with 50 μl of reducing sample buffer. The samples were boiled for 5 min at 95 °C, centrifuged at 13,000 rpm for 5 min and analyzed by SDS-polyacrylamide gel electrophoresis. 25 μl of the sample was used per gel lane. Western blots were then carried out to detect the RHO-GTPases in the samples, using rabbit anti-Rho antibody that reacts with RhoA, RhoB and RhoC, and which is part of the kit. The same antibody was used to measure a total level of RHO-GTPases on the cell extract by western blot in parallel.

### Statistical analysis

Quantitative continuous variables were analyzed using the two-sided Mann–Whitney U test and the Kruskal–Wallis test when more than two groups of samples were compared. Alternatively, two-sided student’s *t* test was used when Gaussian distribution was confirmed. A significance threshold level p = 0.05 was used. Data were analyzed and visualized using GraphPad Prism (GraphPad Software).

## Results

### Aberrant DLC1 DNA methylation in meningiomas

The comparison of meningioma vs. normal meningeal tissue showed significantly higher DNA methylation in tumors at 6 probes clustered at the *DLC1-v1* alternative promoter. These probes represent a differentially methylated region (chr8:13372132-13,373142; hg19). Differences in DNA methylation levels for these probes between tumor and normal samples, measured as the difference of the mean beta-value (proportion of methylated molecules, where 0 denotes lack of DNA methylation and 1 denotes complete methylation), ranged from 0.216 to 0.417. Details are presented in Fig. 1a, b.

Human Methylation 450-K array contains 81 methylation specific probes that span the entire *DLC1* locus. This enables DNA methylation to be compared for the entire gene, including two promoters of other *DLC1* isoforms as well as evolutionary conserved intronic regions. The other *DLC1* alternative promoters were found to be unmethylated in both tumors and control samples. Three additional CpGs at the different intronic regions of *DLC1* locus had significant differences in methylation level. Two of them revealed a slightly decreased DNA methylation level in tumors: cg26148020 (chr8:13136639; hg19) and cg26125690 (chr8:13295113; hg19) with a difference of the mean beta-value 0.270 and 0.227, respectively. One probe showed increased methylation: cg10941185 (chr8:12988517; hg19) with a difference of the mean beta-value 0.345. Generally meningiomas of different grades revealed very similar DNA methylation patterns at the entire *DLC1* locus and no significant differences between tumors of distinct histopathological types were found.

To validate measured differential DNA methylation levels at *DLC1-v1*promoter, two amplicons covering this region were analyzed with single CpG resolution using targeted bisulfite sequencing on a larger group of 50 tumor and 6 normal samples. This confirmed increased DNA methylation in tumors vs. normal meninges and demonstrated very high DNA methylation in the vast majority of tumor samples. The mean DNA methylation levels of 3 CpGs within amplicon 1 were 60 % in meningiomas vs. 19 % in samples of normal meninges (p = 0.0024), whereas the mean DNA methylation level in 5 CpG within the second PCR amplicon were 80 % in meningiomas vs. 37 % in the tissue of meninges (p = 0.0004). Details are presented in Fig. 1d.

### Analysis of DLC1 expression in meningiomas

We selected 18 meningioma tissue samples (4 meningothelial, 4 fibrous, 5 atypical and 6 anaplastic meningiomas) for immunohistochemical staining to detect DLC1 protein expression using a polyclonal antibody. This antibody was produced using immunogen peptide corresponding to N-terminal region of DLC1-v1 and thus it is specific for two isoforms that originate from the promoter region that we found as hypermethylated in meningiomas: DLC1-v1 and DLC1-v3.

**Fig. 2:**
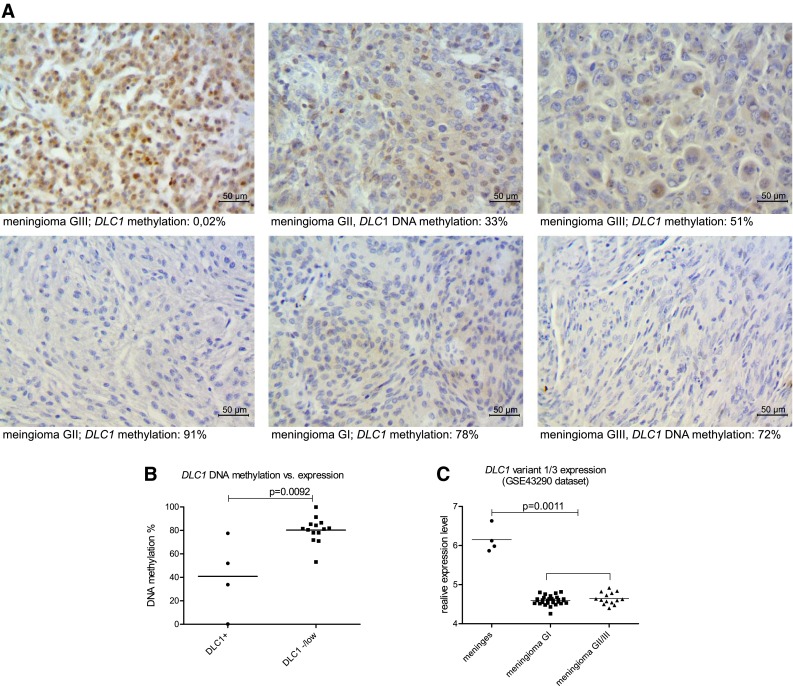
**a** Examples of immunohistochemical staining of DLC1-v1/-v3 protein expression in meningiomas. Magnification ×400. **b** Comparison of DNA methylation levels in meningiomas with high/moderate DLC1 expression and meningiomas with none/poor DLC1 expression. **c** Comparing the relative expression levels of *DLC1v1* and *DLC1v3* measured by 220511_s_at probeset (HG U133A Array, Affymetrix), GSE43290 dataset. *Horizontal lines* represent the mean value

Fourteen tumor Sections (77 %) showed either a lack of, or low DLC1 expression. Moderate or high protein expression was clearly visible in 4 tumor samples: 1 meningothelial (GI), 1 atypical (GII) and 2 anaplastic (GIII) meningiomas and examples are presented in Fig. 2a.

When *DLC1* promoter methylation was matched with expression status, 3/4 expression positive meningiomas showed methylation levels around 50 % or less, whereas 13/14 negative tumor expression showed at least 70 % DNA methylation. When the mean *DLC1* promoter DNA methylation level in expression positive and negative/low samples was compared, significantly lower DNA methylation level in DLC1 immuno-reactive samples was found (mean 40.88 vs 80.29 %, p = 0.0092). This implies a relationship between promoter DNA methylation levels and expression status of corresponding DLC1-v1/-v3 variants in tissue.

We also looked for *DLC1* expression in the available microarray data at the Gene Expression Omnibus. Two data sets, analyzed with two different platforms were used: GSE12530 (CodeLink Human Whole Genome Bioarray, GE Healthcare/Amersham Biosciences, ~45600 genes) and GSE43290 (Human Genome U133A Array, Affymetrix, ~14500 genes). *DLC1* was found in 200 genes most differentially expressed in meningiomas and normal meninges in both datasets. *DLC1* expression levels are significantly downregulated in meningiomas compared to normal meninges, whereas they are comparable in benign and atypical/anaplastic tumors.

We focused on gene annotation for these two particular platforms to verify which *DLC1* expression variants are covered by the differentiating probes. Probes from GSE12530 were described as common for three expression variants 1, 2 and 4 (NM_182643, NM_006094, NM_001164271.1), whereas different Affymetrix probesets from GSE43290 are designed for different *DLC1* variants. This includes two probesets that significantly differentiate meningioma vs. normal meninges; 220511_s_at probeset that cover variants 1 and 3 as well as 210762_s_at probeset that cover exons of variants 1, 2, 4 and 5. Importantly, tumor and meninges samples revealed significantly different signal for the 220511_s_at probeset that cover only the mRNA transcripts originated from the alternative promoter region of DLC1-v1/-v3, that is commonly hypermethylated in meningiomas (Fig. 2).

### Measuring expression levels of different DLC1 variants in meninges and meningiomas

Since it has been previously shown that *DLC1* transcription variants are expressed in a tissue-dependent manner, we designed different sets of PCR primers specific for particular transcripts to measure their expression in normal meninges and meningiomas with qRT-PCR. This analysis included 4 additionally collected sections of snap-frozen normal meninges and 16 meningiomas (15 GI, 1 GII).

**Fig. 3:**
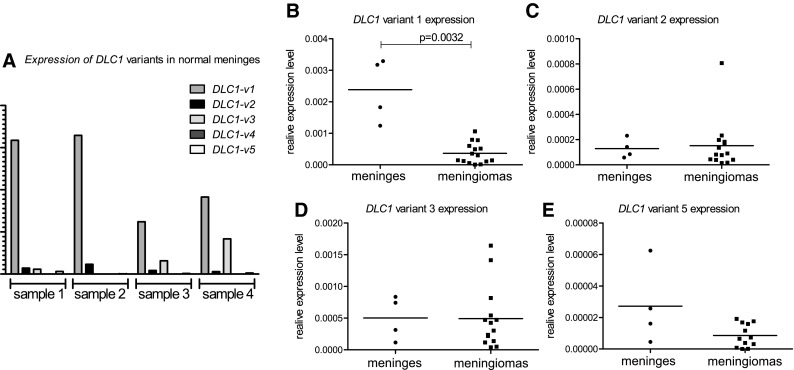
**a** Comparing expression levels of different *DLC1* transcription variants in normal meningeal sections. **b**–**e**: Comparing expression levels of particular *DLC1* isoforms in normal meninges and meningiomas. *Horizontal lines* represent the mean value

Expression levels of *DLC1* variants in normal meningeal samples varied somewhat between individual samples. All gene variants were expressed, except variant 4 which was below qRT-PCR detection limits in nearly all samples. *DLC1-v1* was found to be the most expressed in normal meninges, as shown on Fig. 3. The *DLC1* variant 1 was also the only one to be significantly down-regulated in meningiomas compared to meninges (6.5 fold change of expression p = 0.004), however, a small but insignificant decrease of *DLC1-v5* expression was also observed (Fig. 3). Meningioma samples had slightly variable expression levels of DLC1 isoforms 1, 2 and 3, with an approximately twofold lower expression of DLC1-v2 compared to DLC1-v1 and DLC1-v3, but with an apparently lower RNA expression level of DLC1-v5 (p < 0.0001, Kruskal–Wallis test).

### The role of DLC1-v1 silencing in meningioma cell line

**Fig. 4:**
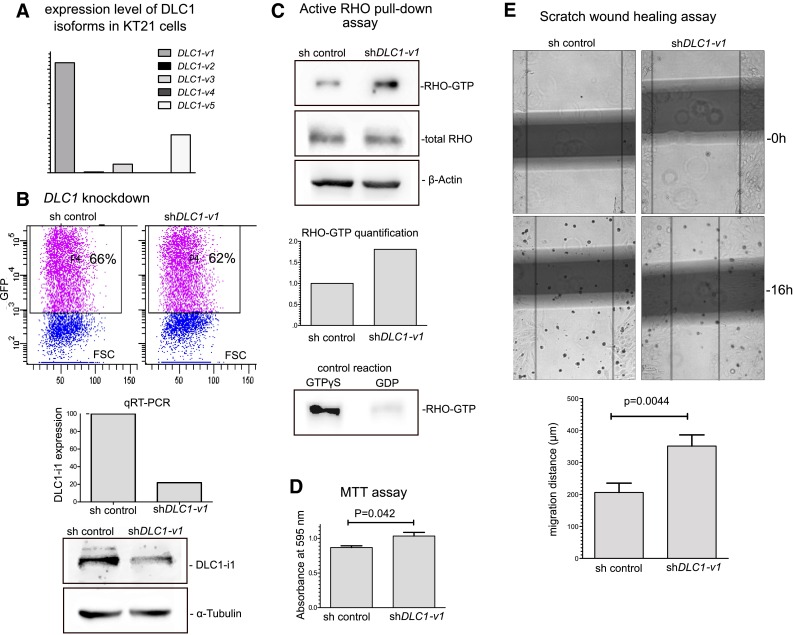
**a** The expression of DLC1 transcription variants in KT21 meningioma cells, qRT-PCR results. **b** Silencing of DLC1-v1 expression in KT21 cells through transfection with plasmid vector containing specific shRNA cassette as compared with cells transfected with control non-effective vector. Cells’ transfection efficacy assessed with flow cytometry, DLC1-v1 mRNA and protein level comparison between KT21 cells transfected with DCL1 specific (sh DLC1-v1) and non-specific shRNA vectors sh control. **c** Active RHO-GTPases pull-down assay in KT21 cells transfected with shRNA targeting DLC1 and control cells. RHO-GTPases were detected with antibody specific to RhoA, RhoB and RhoC. **d** Result of MTT test performed in KT21 cells transfected with shRNA targeting DLC1 and control cells. **e** The results of woundhealing assay performed in KT21 cells transfected with shRNA targeting DLC1 and control cells. The examplary fields marked for the measurement of the migration distance. The comparison of migration distance of shDLC1-1 and sh-control KT21 cells.

To investigate the role of *DLC1-v1*downregulation we performed a knockdown experiment in KT21 cell line established from human malignant meningioma [16]. Initially, we determined the expression level of different *DLC1* variant in KT21 cells. DLC1-v1 appeared to be the predominant one, however DLC1-v5 also was found expressed in these cells (Fig. 4).

KT21 cells were transfected with plasmid vector containing shRNA cassette targeting 5′ region of DLC1-v1/-v3 using lipofectamine 2000, to decrease the expression level of these transcription variants. Control cells were transfected with the same vector type with non-effective shRNA cassette (sh control), available commercially. In the initial experiment transfected cells were analyzed by flow cytometry that showed relatively good transfection efficacy ~60 % and sorted with cell sorter using GFP fluorescence. The effect of transfection with shRNA plasmid was tested on sorted cells. This revealed a decrease of *DLC-v1* mRNA level up to 22 % of the expression level in control cells as well as visible protein level downregulation (Fig. 4).

Three experiments were performed on KT21 cells after transfection with plasmid vector targeting *DLC1* expression to investigate the effect of gene downregulation on selected biological processes that are related to a known DLC1 activity: activation of RHO-GTPases, cell metabolic activity and cell migration. As observed in active RHO-GTPases pull down assay transfection of KT21 cells with shRNA targeting DLC-v1 resulted in the increased activity of RhoA, RhoB and RhoC as compared to control cells (Fig. 4.). As RHO-GTPases are known regulators of cell migration, particularly through ROCK kinase pathway this result is in line with the outcome of the cell migration test. KT21 cells treated with shRNA against DLC-v1 where characterized by increased migration. These cells covered the cell-free area in the woundheeling scratch assay significantly more quickly than control cells (p = 0.0044) (Fig. 4). Transfection with shDLC1-v1/-v3 resulted also in the slightly higher metabolic activity of the cells as observed in MTT test (p = 0.042).

## Discussion

Downregulation of *DLC1* in meningiomas was previously shown in a small study on 6 tumor samples. Importantly, this gene acts as a tumor suppressor and the transfection of primary meningioma cultured cells with normal DLC1 results in a decrease of meningioma cell growth. A search of deposited microarray expression results also showed decreased *DLC1* levels in meningiomas compared to normal meninges in different publicly accessible datasets. These preliminary data supported the idea that *DLC1* is a tumor suppressor that is silenced in meningiomas, similar to other human tumors such as nasopharyngeal, pancreatic, colon, prostate and liver cancer [4, 6, 17–19].

*DLC1* downregulation in tumors can arise from genomic mutation or be due to aberrant epigenetic regulation including high DNA methylation levels at the gene promoter region, as it is frequently observed in various tumors [6, 17–19].

Interestingly, a previous study on *DLC1* downregulation in meningiomas demonstrated a lack of promoter DNA hypermethylation in tumors. It was stated that the gene is not inactivated via DNA methylation mechanism [10]. In our study, we identified *DLC1* as being frequently hypermethylated in meningiomas from genome-wide comparisons of different meningiomas and normal meninges sections. At first glance this may seem to be contrary to previous report, however, it appears that the complex structure and regulation of the *DLC1* genomic loci can explain this inconsistency.

The *DLC1* gene encodes 5 different protein isoforms and gene transcription can originate from different promoter regions (Fig. 1a). This regulation mechanism is common to all DLC family members of RHO GTP-ases and different identified isoforms of *DLC2* (*STARD13*) and *DLC3* (*STARD8*) are transcribed from alternative promoters. *DLC-1* isoforms, except from *DLC1-v3* [7], encode functional RHO GTPases that differ in N-terminal region and share the same functional domains [3]. It seems that they differ in subcellular localization. DLC1-v1and DLC1-v2 are found at focal adhesion sites, DLC1-v3 in the cytoplasm, whereas DLC-i4 is a putative mitochondrial protein [7, 9]. DLC-v2 was also observed in adherence junctions, cytosol and the nucleus [20–22]. The exact differences in biological roles of the different isoforms remain elusive, however, tumor suppressor activity was confirmed for different isoforms encoding functional RhoGAP domain [7, 9].

Previous studies on *DLC1* isoforms 1–3 and 5 showed that they are all expressed in various human tissues, including brain [9]. However, actual expression levels of different gene isoforms seem to be tissue dependent [7, 8]. Human spleen, liver and colon tissues show the highest level of *DLC1* isoform 2, the thyroid shows comparable levels of isoforms 1 and 2, whereas nearly exclusive expression of isoform 1 was found in the heart [7].

The majority of cancer studies on *DLC1* silencing, does not address the problem of the complex gene transcription nor the expression of alternative isoforms, but are instead focused on the expression of the gene variant 2 or they utilize the qRT-PCR primers or antibodies not specific to given isoforms. The expression of different *DLC1* isoforms was determined in hepatocarcinoma, where *DLC1-v2*, but not *DLC1-v1* downregulation was observed [7]. In another study, frequent epigenetic downregulation of *DLC1-v5* was identified in certain human cancers and a panel of different cell lines [9].

Microarray data from previously performed expression profiling of meningiomas and normal meninges, with Affymetrix U133 expression arrays (GEO GSE43290), showed that significantly decreased signal occurred in tumor samples for a probeset specific to *DLC-v1* and *DLC1-v3* isoforms that share the unique N-terminal region. These two isoforms are transcribed from the promoter region which we observed to be hypermethylated in meningiomas.

To determine the potential relationship between the promoter methylation of the DLC1 promoter that we found with expression of *DLC1-v1* and *DLC1-v3* isoforms we performed a series of immunohistochemical staining of meningiomas’ sections with the antibody specific to N-terminal region of DLC1-v1/-v3 isoforms. As clarified with the supplier of this antibody, it does not detect the remaining DLC1 protein isoforms. Low or no DLC1 protein expression was observed in a large proportion of tumor tissues, in accordance with the mentioned microarray results. Additionally, a relationship between tissue immunoreactivity and DNA methylation was observed as the samples with positive DLC1-v1/-v3 staining showed significantly lower methylation level of the corresponding promoter region than DLC1 negative ones.

The above findings indicate that isoforms DLC1-v1/-v3 are downregulated in DNA methylation dependent manner in meningiomas and suggested that these isoforms may be substantially relevant in meninges and their tumorigenesis. When we compared mRNA expression levels of 5 different DLC1 isoforms in normal meninges, it appeared that *DLC1-v1* was the one most expressed. This isoform was also the only one that is significantly downregulated in meningeal tumors. We did not observe any such significant downregulation of *DLC-v2*, that is highly expressed transcription variant in most of previously investigated tissue types.

The promoter region of *DLC1-v1*/*v3* have been previously described. The binding sites of particular transcription factors, including TP53, STATs, HSF and E2F, were identified and promoter activity of this region was confirmed by the luciferase reporter assay [9]. The epigenetic DNA methylation dependent regulation of this promoter was considered as rather unlikely, because the region does not meet the criteria of CpG island and the expression of *DLC1-v1* was not restored after 5-aza-2′-deoxycytidine treatment in selected cell lines [7, 9]. However, it is worth noting that the exact DNA methylation pattern of these regions was not determined in cell lines and tissue samples, so it is not clear if resumption of gene expression could be expected in the tested cells by targeting DNA methylation. It is also seen that, in general, DNA methylation of non-CpG island gene promoters can affect gene expression [23, 24].

Because DLC1-v3 lack the functional domains it is considered as nonfunctional or putative regulatory protein [7]. In turn, DLC1-v1 that is fully active protein, significantly downregulated in meningiomas turns out to be the most relevant form of DLC1 protein in neoplastic transformation of meninges. The onco-suppressive role of this protein variant was shown previously in hepatocellular carcinoma (HCC) cell lines. DLC1-v1 inhibited stress fibre formation and suppressed growth of HCC cells [7]. In another study, the overexpression of this variant in HCT116 colorectal carcinoma cells and KYSE510 cells from esophageal cancer resulted in suppressed tumor cell colony formation [9]. Together this is concordant with the results of functional analysis of the mutations that were found in N-terminal domain of *DLC-v1* in patients with sporadic congenital heart disease. These mutations, disturbing DLC1 structure, appeared to increase cell migration of human umbilical vein endothelial cells (HUVEC) and human bone marrow endothelial cells 60 (HBMEC-60) [25]. In our hands, targeting of *DLC1-v1* with specific shRNA in KT21cells established from human malignant meningioma resulted in enhanced activity of RhoA/RhoB/RhoC GTPases as shown in active RHO pull down assay and increased cell migration in scratch assay. This results corroborates the previous report and confirms that DLC1-v1act as tumor suppressor.

DLC1 regulates cell migration through the inhibition of RHO-GTPases/ROCK kinase signaling as well as in RHO-independent manner. Both pathways lead to the cytoskeletal reformation and the changes of focal adhesion and intercellular junctions, especially adherence junctions maintained by cadherins [3]. Interestingly, in line with the observation of the aberrant DLC1 expression in meningiomas, both focal adhesion and adherence junction formation processes were found among the most disturbed in meningioma pathogenesis and progression [26, 27].

In summary, our results show for the first time that CpGs of the *DLC1-v1* alternative promoter is frequently hypermethylated in tumors of meningeal origin. Significantly higher methylation levels of this region was observed in meningiomas without *DLC-1* protein expression compared to immunoreactive tumors. The remaining *DLC1* alternative promoters are unmethylated. This includes the promoter of *DLC1-v2* that was reported as being hypermethylated in diverse cancers. This result is consistent with the previous report showing lack of *DLC1* methylation in meningiomas, because it was focused on the DNA methylation at the promoter of *DC1-i2* only [10].

Additional comparison of mRNA expression levels of all known *DLC1* isoforms showed that *DLC1-v1* is the one most expressed in normal meninges and its expression level is most notably reduced in meningiomas. This allows for a conclusion that in meningiomas, the decreased DLC1 isoform 1, (with proven suppressor activity), contributes to tumorigenesis and that the DNA methylation of the corresponding promoter regions may play a role in gene silencing. We believe that our study indicates a promising direction for further research, because different transcription variants of many known tumor-related genes can be regulated via alternative promoters. The well-known examples are *TP53, TP63, TP73, CDKN1A, CDKN2A* or *PTCH1* [28, 29]. In general, the role of abnormal epigenetic regulation of alternative gene promoters in cancer is poorly understood.
